# Molecular analysis of cryptosporidiosis on cattle farms in Gran Canaria, Canary Islands (Spain)

**DOI:** 10.1080/23144599.2025.2460923

**Published:** 2025-02-10

**Authors:** María Cristina Del Río, Sergio Martín, Joaquín Quílez, Claudia Vergara-Castiblanco, José Manuel Molina, Otilia Ferrer, Magnolia María Conde, José Adrián Molina, Antonio Ruiz

**Affiliations:** a Parasitology Unit, Department of Animal Pathology, Faculty of Veterinary Sciences, University of Las Palmas de Gran Canaria (ULPGC), Arucas, Las Palmas, Spain; b Medical and Surgery Unit, Department of Animal Pathology, Faculty of Veterinary Sciences, University of Las Palmas de Gran Canaria (ULPGC), Arucas, Las Palmas, Spain; c Parasitology Unit, Department of Animal Pathology, Faculty of Veterinary Sciences, University of Zaragoza, Zaragoza, Spain; d Parasitology Unit, Department of Animal Health, Faculty of Veterinary Sciences, University of Córdoba, Córdoba, Spain

**Keywords:** *Cryptosporidium*, bovine, subtyping, risk factors, zoonosis, Canary Islands

## Abstract

*Cryptosporidium* spp. infections in calves cause serious economic losses in livestock and pose an important zoonotic risk. The aim of this study was to investigate the prevalence of *Cryptosporidium* spp. in cattle on the island of Gran Canaria. Faecal samples were collected from calves and adult cattle from a total of 15 farms, and a questionnaire survey was conducted to farmers. The presence of *Cryptosporidium* spp. oocysts in faeces was determined by microscopy, showing infection rates of 45.9% in calves and 4.1% in adults, with positive correlation with the faecal scores of infected animals (*p* < 0.0001). Samples were amplified by PCR targeting SSU rRNA, with positivity rates for calves and adults being 51.7% and 31.7%, respectively. The PCR-positive samples were further genotyped and sequenced for the 60 kDa glycoprotein gene (GP60) and the microsatellite TP14. Four *Cryptosporidium* species were identified (*C. parvum, C. ryanae*, *C. bovis,* and *C. andersoni*), of which *C.* *parvum* was the most frequent in calves (45.8%) and adults (29.2%). GP60 sequencing revealed that all *C. parvum* samples belonged to the IId family, the most frequent subtypes being IIdA22G1 and IIdA23G1. Overall, the results of this study demonstrate a high occurrence of *Cryptosporidium* spp. in both calves and adult cattle, including the zoonotic IId family of *C. parvum*. These findings have significant implications for cattle farming and public health. The lack of awareness among farmers regarding cryptosporidiosis highlights the need for caution to prevent epidemiological outbreaks that could impact both human and livestock health.

## Graphical abstract

**Figure:**
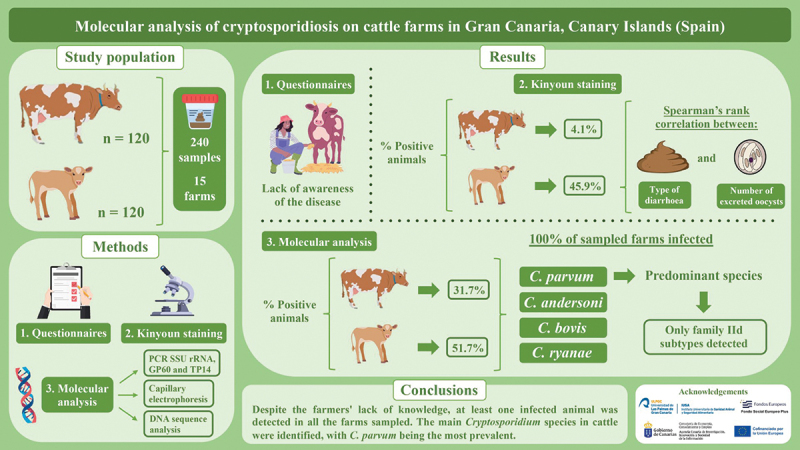

## 1. Introduction

*Cryptosporidium* spp. are protozoan parasites from the phylum Apicomplexa that cause gastrointestinal diseases affecting multiple animal hosts, including mammals, birds, amphibians, and reptiles [1,2]. In young ruminants, cryptosporidiosis causes great economic losses as there is currently no fully effective treatment against this parasitic infection [3]. Cryptosporidiosis in calves typically occurs between the first and third week of life by direct oral transmission through ingestion of oocysts present in the faeces of infected animals, or by indirect transmission through ingestion of water or food contaminated with oocysts. The peak of oocyst excretion is reached 5–6 days post infection, and as the animal grows older the number of oocysts is excreted and the severity of symptoms decreases. The most frequent symptoms include watery or bloody diarrhoea, loss of appetite, anorexia, pain, apathy, growth retardation, and weight loss. Differences in severity of symptomatology between calves have been associated with host immune status, co-infections with other pathogens and different species as well as genotypes of *Cryptosporidium* [4–8].

So far, four *Cryptosporidium* spp. infecting cattle have been described, with differences in pathogenicity and in the age range of the animals they affect. *C. parvum* is more frequent in suckling calves, *C. bovis* and *C. ryanae* in weaned calves, and *C. andersoni* in yearlings and adult cattle. *C. parvum, C. bovis* and *C. ryanae* have been shown to cause villi atrophy, microvilli shortening and intestinal damage, whereas *C. andersoni* primarily affects the abomasum, leading to gastritis. Cattle infections with *C. ryanae* and *C. bovis* have been associated with asymptomatic infections, while *C. andersoni* is implicated in chronic infection and does not produce diarrhoea [4,7,9,10].

Determination of the different species of *Cryptosporidium* is important for taxonomic, diagnostic and epidemiological purposes, and stains such us Kinyoun, Heine and fluorochrome (Auramine) are characterized as inexpensive diagnostic techniques that allow preliminary detection of the parasite [8]. However, the low parasite-host specificity, along with the difficulty in stablishing morphometric differences in oocysts among *Cryptosporidium* spp., has prompted the development of molecular techniques as alternatives to microscopic identification for *Cryptosporidium* spp. [11–14]. Some of the highly preserved genes commonly used include the oocyst wall protein (COWP), heat shock − 70kDA protein (HSP70), the gene encoding actin and the gene of the small-subunit of ribosomal RNA (SSU rRNA), the latter being a widely used marker as it contains multiple conserved regions of *Cryptosporidium* genus that enable the development of primers for most *Cryptosporidium* species [15–18].

On the other hand, variable sequences of less-conserved genes have been used in population genetic studies to identify families of *Cryptosporidium* subtypes, which has permitted epidemiological studies of *C. parvum* to be carried out, the identification of zoonotic-specific subtypes and tracking possible sources of infection [11,19,20]. The 60 kDa glycoprotein gene (GP60) is one of the most polymorphic markers identified so far in the *Cryptosporidium* genome. Sequencing of this gene has revealed at least 14 families among *C. parvum* isolates from humans and/or cattle (IIa to IIo) and several subtypes within each family [4]. Some of *C. parvum* families can affect both humans and ruminants (IIa and IId), while others are more specific to humans (IIc) [21]. In many countries, family IIa has been identified mainly in calves, with subtype IIaA15G2R1 being the most predominant, while family IId has been more commonly associated with lambs and goat kids [14,20,22,23]. On the other hand, microsatellites, also called short variable number tandem repeat (VNTR), are fragments with short tandem repeats of base pairs whose amplification by PCR and separation of the fragments by length allows a better understanding of the genetic differences or similarities between two individuals. More than 50 mini- and microsatellites have been used in previous studies for multilocus fragment typing (MLFT), of which TP14 provides the best discrimination between *C. parvum* and *C. hominis* [23–27].

Only a few studies on *Cryptosporidium* have been performed in the Canary Islands, where the parasite has been detected in wastewater [28], human faecal samples [29], birds [30], hedgehogs [31,32], rabbits [33] and rodents [34]. As there are no previous studies on cryptosporidiosis in either large or small ruminants, the present study constitutes the first molecular and epidemiological analysis of bovine cryptosporidiosis in the Canary Islands. Livestock farming is a key sector in the region, and cryptosporidiosis could pose a threat to the development of profitable and sustainable canary dairy cattle farms. Furthermore, this issue would raise public health concern if the presence of zoonotic *Cryptosporidium* species and subtypes is confirmed in cattle. For all these reasons, molecular and epidemiological studies to detect the presence of this parasite on cattle farms as a preliminary step towards the implementation of control measures are needed. So that, the main objectives of this study were to provide data on the occurrence of *Cryptosporidium* spp. infections in dairy cattle from farms located in different municipalities of the island of Gran Canaria, their subsequent molecular characterization of *C. parvum* isolates using GP60 subtyping and the TP14 marker, and to conduct an analysis of the main risk factors associated with cryptosporidiosis through questionnaire surveys.

## 2. Materials and methods

### 2.1. Sample collection

**Figure 1.**
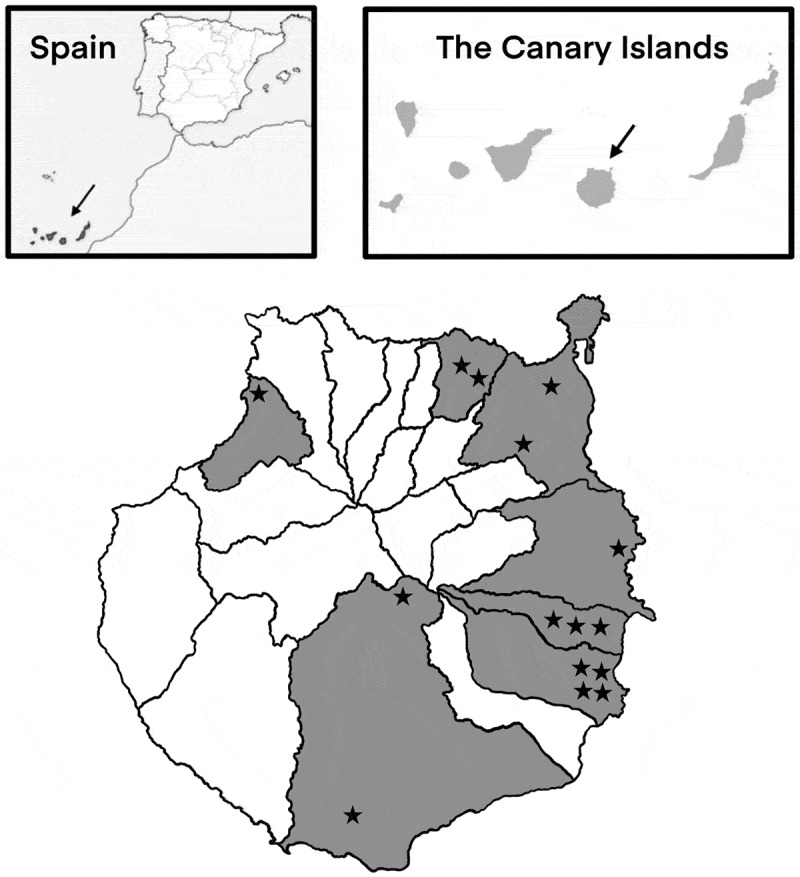
Geographical distribution of the 15 farms sampled on the island of Gran Canaria (Spain).

The sample collection for the present study was conducted in seven municipalities of the island of Gran Canaria (Canary Islands, Spain) (Figure 1), over a period of 4 months. A total of 15 dairy cattle farms, varying in size, production systems, management, and hygienic-sanitary measures, were included in the survey. From each farm, eight faecal samples were collected from calves 1–2 weeks old, as well as from eight recently calved adult cows. The number of animals sampled per farm was determined through statistical calculations using WinEpiscope software (http://www.winepi.net/). Faecal samples were obtained by digital stimulation, directly from the rectum, to prevent contamination. The faeces were collected in sterile, properly labelled tubes and a faecal score was established according to their consistence: (1) normal faeces/no diarrhoea, (2) pasty faeces, (3) loose faeces, (4) liquid faeces, and (5) liquid faeces with blood or intestinal mucosa. The samples were then refrigerated in a cool box and taken to the Faculty of Veterinary Medicine at the University of Las Palmas de Gran Canaria (ULPGC), where they were stored at 8°C until further processing. Overall, a total of 240 faecal samples, including 120 samples from calves and 120 samples from adult cows, were collected during the study. Experimental procedures have been approved by institutional review board-approved protocols (reference: OEBA-ULPGC-37/2024).

### 2.2. Microscopic analysis

Following an ether sedimentation concentration technique, the presence of *Cryptosporidium* oocysts in the faecal samples was detected using Kinyoun staining and subsequent microscopic examination [35]. Briefly, one drop of the sediment was smeared onto a slide, allowed to dry and fixed with methanol for 1 min. The sample was then stained with carbol fuchsin for 2 min, followed by rinsing with 50% ethanol for 1 min. Decolorization was performed using 1% sulphuric acid for 20 s, and methylene blue was applied for 1 min.

To evaluate the parasitic load of each sample, an approximate estimation of the *Cryptosporidium* oocyst counts (Estimation Oocyst Count – EOC) was performed. For that, each sample was examined under a microscope at 1000X magnification for exactly 10 min. The examination and oocyst counting were conducted in the areas of the smears with the highest staining quality, typically near the margins of the smear. As a rule, oocyst counts were conducted across 50–80 randomly selected fields, except in cases of very high parasitic load, where counts were limited to 25 random fields. The infection intensity in each sample was classified as follows: high intensity (>25 oocysts), moderate intensity (16–25 oocysts), mild intensity (6–15 oocysts), minimal intensity (1–5 oocysts) and no infection (0 oocysts).

### 2.3. Molecular analysis

#### 2.3.1. DNA extraction

Aliquots of the faecal sediment from all 240 samples were kept at 4°C for subsequent DNA extraction. Total DNA was extracted from 200 µl of the concentrated sediments by three freeze–thaw cycles to facilitate the rupture of oocyst walls. During each cycle, samples were frozen in liquid nitrogen for 1 min and then incubated at 100°C for 5 min, following a method previously described [18,36]. DNA extraction was performed using the QIAamp DNA mini kit (Qiagen®, Germany) according to the manufacturer’s instructions.

#### 2.3.2. PCR primers and conditions

Each sample was subjected to the following: (1) a nested PCR to amplify a fragment of the SSU rRNA gene, (2) a single PCR to amplify a fragment of the 60-kDa glycoprotein gene (GP60), and (3) a nested PCR to amplify the TP14 microsatellite.

All primer sequences used have been previously described (Table 1). To identify overlapping peaks in the capillary electrophoresis analysis, the reverse primers in the single PCR and the secondary reverse primers in nested PCRs were labelled with different fluorophores: TAMRA (6-carboxytetramethylrhodamine), HEX (4,7,2“,4”,5“,7”-hexachloro-6-carboxyfluorescein) and FAM (6-carboxyfluorescein).

**Table 1.** Primers used for the different locus and predicted fragment size range of PCR-amplified products.

Locus	Primer	Primer sequence	Fragment size range (bp)	Reference
SSU rRNA	F1	5′-GGAAGGGTTGTATTTATTAGATAAAG- 3′	386–399	Ramo et al. [18]
	R1	5′-AAGGAGTAAGGAACAACCTCCA- 3′		
	F2	5” -AATTGGAGGGCAAGTCTGGT- 3”		
	R2-TAMRA	5′- AACATCCTTGGCAAATGCTT-3′		
GP60	F	5′-CCAGCCGTTCCACTCAGA- 3′	333–366	Ramo et al. [18]
	R-HEX	5′-GGTACCTTCTCCGAACCACA- 3′		
TP14	F1	5” -TAATGCCCACCCATCTTCTT- 3”	279–333	Quílez et al., [37]
	R1	5” -TCCATCTGGGTCCATTTAGC- 3”		
	F2	5” -CTAACGTTCACAGCCAACAGTACC- 3”		
	R2-FAM	5” -GTACAGCTCCTGTTCCTGTTG- 3”		

In the nested PCR used to amplify the SSU rRNA gene, identical reaction mixtures were prepared for both the primary and secondary PCRs. The PCR mixture consisted of 3 mm MgCl_2_, 1X PCR buffer, 200 μM deoxynucleoside triphosphate (dNTPs), 0.2 μM of each primer, 0.1 mg/ml bovine serum albumin (BSA), 1 U Taq polymerase (iNtRON Biotechnology, Republic of Korea) and 2 μl of DNA template (for primary PCR) or 2 μl of primary PCR product (for secondary PCR) in a total reaction volume of 20 μl. The primary PCR was performed with an initial denaturation step at 94°C for 3 min, followed by 35 cycles consisting of denaturation at 94°C for 45 s, annealing at 55°C for 45 s, and extension at 72°C for 1 min, with a final extension at 72°C for 7 min. The same protocol was applied for the secondary PCR, except that the annealing temperature was adjusted to 60°C for 45 s.

For the single PCR targeting the GP60 gene, the PCR mix consisted of 1.5 mm MgCl_2_, 1X PCR buffer, 200 μM dNTPs, 0.5 μM of each primer, 0.1 mg/ml BSA, 1 U Taq polymerase (iNtRON Biotechnology, Republic of Korea) and 2 μl of DNA template in a total reaction volume of 20 μl. This reaction was subjected to an initial denaturation step at 94°C for 5 mins, followed by 40 cycles of 94°C for 30 sec, 62°C for 30 s, and 72°C for 1 min, with a final extension at 72 º for 7 min.

As for the nested PCR amplifying the TP14 microsatellite, different protocols were assessed for the primary and secondary PCRs. The primary PCR mix consisted of 2.5 mm MgCl_2_, 1X PCR buffer, 200 μM dNTPs, 0.5 μM of each primer, 0.1 mg/ml BSA, 1 U Taq polymerase (iNtRON Biotechnology, Republic of Korea) and 1 μl of DNA template in a total reaction volume of 10 μl. The secondary PCR mixture consisted of 2.5 mm MgCl_2_, 1X PCR buffer, 200 μM dNTPs, 0.5 μM of each primer, 0.1 mg/ml BSA, 1 U Taq polymerase (iNtRON Biotechnology, Republic of Korea) and 2 μl of primary PCR product in a total reaction volume of 20 μl. The resulting primary PCR TP14 mix was subjected to an initial denaturation step at 95°C for 3 min, followed by 35 cycles of three steps (95°C for 50 s +61°C for 50 s +72°C for 1 min), and a final extension at 72°C for 10 min. We performed the same protocol in the secondary PCR except for the second step within the 35 cycles: 62°C for 50 s.

PCR products were separated by electrophoresis on a 1.5% agarose gel, and DNA amplification was confirmed by visualization with GelRed nucleic acid gel stain (EMD Millipore Corp., USA).

#### 2.3.3. Capillary electrophoresis (CE)

A total of 183 PCR-positive samples with clearly visible bands on gel electrophoresis were selected for capillary electrophoresis. Based on the amplicon intensity, between 1 and 3 μl samples of the SSU rRNA, GP60 and TP14 PCR-amplified products from each *Cryptosporidium* isolate were mixed to a final volume of 6 μl. These samples were then subjected to CE using a 3500×L Genetic Analyzer and sized automatically making use of the GeneScan 600 Liz Size Standard and the Gene Mapper Software (Applied Biosystems, Life Technologies). For each isolate, a multicolour electropherogram was generated, showing three fluorescence peaks corresponding to the length of the PCR product for each marker. An allele was assigned based on the fluorescence colour and predicted amplicon size. The presence of two distinct peaks for a specific locus, differing by multiples of the repeat unit, was interpreted as an indication of a mixed infection.

#### 2.3.4. DNA sequence analysis

Isolates representing different alleles at each locus were selected and sequenced to identify potential size differences and to establish their identity with alleles determined by CE analysis. The PCR products were purified and sequenced in both directions on a 3500×L Genetic Analyser (Applied Biosystems®, Life Technologies) according to the manufacturer’s instructions. The sense and antisense strand sequences were aligned using CLUSTAL W and edited with MEGA 11.0.13 (https://megasoftware.net). Consensus sequences were analysed using BLASTN searches in the NCBI databases (http://blast.ncbi.nlm.nih.gov/Blast.cgi). Representative nucleotide sequences generated in this study were deposited in the GenBank database under the following accession numbers: PP177439, PP177440, PP177441, PP177444, PP213048, PP213049, PP333105, PP333106, PP333107, and PP333108.

#### 2.3.5. Phylogenetic analysis

The sequences of the alleles obtained were compared with the GenBank database, assigning each allele of the SSU rRNA gene to a *Cryptosporidium* species and the alleles of the GP60 gene to a group of *Cryptosporidium* family. TCA and TCG repeats in the trinucleotide repeat region and mutations in the non-repeat regions determined the subtype within each *Cryptosporidium* family [38].

Phylogenetic analysis was performed using MEGA11.0.13 software. Neighbour-Joining trees were constructed considering the evolutionary distances calculated using Kimura two-parameter model. For greater reliability of the trees, bootstrap analysis with 1000 replicates was used, discarding values below 50%. Neighbour-joining SSU rRNA, GP60 and TP14 trees were rooted with *Plasmodium cathemerium* (AY625607.1), *C. parvum* IIcA5G3a (AY738195.1) and *C. hominis* (MT118812.1) sequences, respectively.

### 2.4. Questionnaire

To identify risk factors and estimate the economic losses associated with cryptosporidiosis in Canarian dairy cattle farms, livestock farmers were interviewed using a questionnaire after sample collection. For more technical questions, veterinarians were also surveyed. The questionnaire comprised 21 questions covering different topics: farm/farmer information (1/21), cattle breed (1/21), facilities and management practices (7/21), knowledge about cryptosporidiosis (1/21), treatments used for neonatal diarrhoea and their effectiveness (3/21), clinical signs and outcomes of cryptosporidiosis (6/21) and direct or indirect costs associated with *Cryptosporidium* spp. infections (2/21).

Since all farms tested positive for the SSU rRNA and no quantification of the amplicons was made by RT-PCR, molecular identification of this gene marker could not be used as discriminating factor. Instead, parasitic load-EOC based on Kinyoun staining results was employed. Additionally, the number of positive animals per farm, as determined by either Kinyoun staining or SSU rRNA gene amplification, was used to assess their association with the variables surveyed in the questionnaire.

### 2.5. Statistical analysis

A table was created in Microsoft Excel® to record data on faecal scores, EOCs and PCR positivity for SSU rRNA. To compare results between farms a Kruskal–Wallis one-way analysis of variance on ranks was used. Due to the large variation in EOC (*n*) within the same farm, the results were normalized by Log10 (*n* + 1). A correlation analysis was performed using Spearman’s rank correlation test to examine the relationship between the number of oocysts excreted and the faecal consistency observed in the animals. Finally, Square Chi Test was employed to compare the number of positive samples between calves and adults, and microscopy versus SSU rRNA gene amplification. All analyses were conducted using Sigmaplot 14.5 software, with statistical significance set at a *p* < 0.05.

The questionnaires were digitized in Microsoft Excel® to identify risk factors for cryptosporidiosis in calves and cows. The EOC results and the number of positive animals per farm were compared to the various items assessed in the questionnaire. The results from the different farms surveyed were compared using dynamic tables and graphically represented to facilitate the interpretation of potential correlations. The statistical analysis was performed by the Fisher's exact test by using the same software and statistical significance.

## 3. Results

### 3.1. Microscopic analysis and faecal scoring

**Figure 2.**
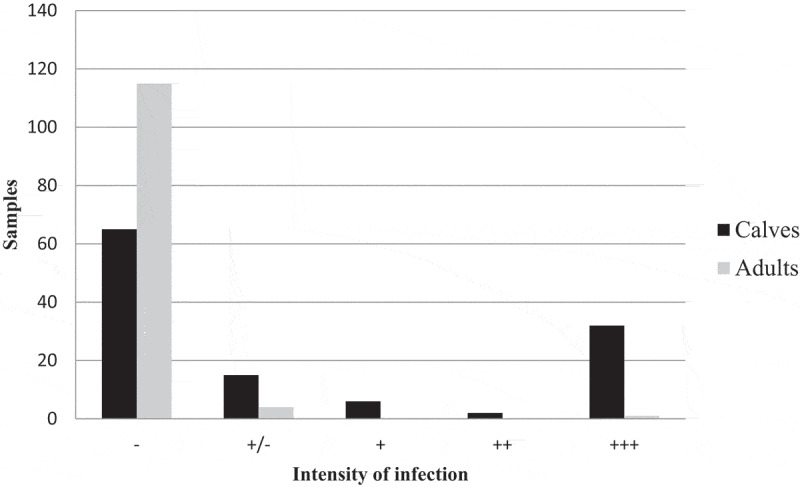
Comparison of the intensity of infection in calves and adults detected by Kinyoun staining.

A total of 45.9% (55/120) Kinyoun stain positive faecal smears were recorded in calves in 14 out of the 15 farms sampled (93.3%). Mean and standard error of oocyst estimation counts (EOC) and faecal scores per farm are shown in Table 2. A high parasitic load (>25 EOC) was observed in 26.7% of the positive calf samples, with more than 5,000 *Cryptosporidium* spp. oocysts being counted in 25 fields from a single sample. Positive calf samples with moderate (16–25 EOC), mild (6–15 EOC) or minimal (1–5 EOC) infection intensity were found in 1.7%, 5%, and 12.5% of the calves, respectively, while 54.1% of the calves tested negative by Kinyoun staining. In adult cows, only 4.1% of (5/120) samples from three farms tested positive by microscopy. In most positive samples, one to five *Cryptosporidium* spp. oocysts were observed, with only one sample showing a higher count (34 oocysts). The percentage of animals with different intensities of infection stated was as follows: 0.8% (high), 0% (moderate), 0% (low), 3.3% (minimal), and 95.9% (negative) (Figure 2).

**Table 2.** Means and standard errors of estimation oocyst counts (EOC) and faecal score per farm.

	Calves		Adults	
Study farms	EOC	Faecal score	EOC	Faecal score
F1	17.1 (±12.5)	2.4 (±0.2)	0.0 (±0.0)	1.0 (±0.0)
F2	0.7 (±0.5)	2.8 (±0.3)	1.1 (±0.8)	1.0 (±0.0)
F3	15.9 (±13.4)	2.9 (±0.5)	0.0 (±0.0)	1.0 (±0.0)
F4	275.1 (±292.7)	3.1 (±0.3)	4.3 (±4.5)	1.0 (±0.0)
F5	45.8 (±27.4)	3.3 (±0.3)	0.0 (±0.0)	1.0 (±0.0)
F6	49.1 (±32.9)	2.4 (±0.5)	0.0 (±0.0)	1.0 (±0.0)
F7	62.8 (±62.3)	2.1 (±0.1)	0.0 (±0.0)	1.0 (±0.0)
F8	989.9 (±674.6)	2.6 (±0.4)	0.0 (±0.0)	1.0 (±0.0)
F9	136.6 (±84.3)	3.0 (±0.3)	0.0 (±0.0)	1.0 (±0.0)
F10	28.3 (±23.8)	2.4 (0.3)	0.4 (±0.3)	3.0 (±0.0)
F11	0.0 (±0.0)	2.3 (±0.4)	0.0 (±0.0)	1.0 (±0.0)
F12	13.6 (±14.6)	2.9 (±0.1)	0.0 (±0.0)	1.0 (±0.0)
F13	0.5 (±0.4)	2.4 (±0.3)	0.0 (±0.0)	1.0 (±0.0)
F14	52.1 (±48.1)	3.0 (±0.3)	0.0 (±0.0)	1.0 (±0.0)
F15	72.9 (±66.1)	2.6 (±0.4)	0.0 (±0.0)	1.0 (±0.0)

Of the 15 farms sampled, 8.3% calves from five farms showed no signs of diarrhoea. Faecal scores indicating pasty (2) and loose faeces (3) were recorded in 37.2% and 36.3% of calves, respectively. Liquid diarrhoeas (4) were observed in 17.4% of calves from 10 farms, while a single calf was classified with the highest faecal score (5). Among adult cattle, 93.3% of samples showed no signs of diarrhoea, with only one farm reporting faeces classified as “3” (6.7%), which the farmer attributed to a dietary change.

In calves, 96.4% (53/55) of the Kinyoun stain positive samples also had diarrhoea, while in adults only 40% (2/5). Spearman’s rank order correlation analysis confirmed a statistically significant relationship between the type of diarrhoea and the oocyst estimation counts, with a correlation coefficient of 1.000 and a *p* < 0.0001.

### 3.2. Molecular analysis

The occurrence of different alleles that could be correctly identified by CE for each locus, along with their corresponding frequencies across the 15 farms sampled, are shown in Table 3. A total of 51.7% (62/120) of calves and 31.7% (38/120) of adults tested positive based on the SSU rRNA gene marker results, with significant differences being recorded between these two age ranges (*p* < 0.01). Molecular detection of *Cryptosporidium* oocysts in calves did not significatively increase the sensibility of Kinyoun staining, but statistically higher positivity was found in adult samples with the SSU rRNA gene marker than by microscopical analysis (*p* < 0.001). On the other hand, in calves, 96.8% (60/62) of the SSU rRNA-positive animals also had diarrhoea, while in adults only 5.3% did (2/38). Positive samples from both calves and adults were detected in 100% of the farms tested.

**Table 3.** Allele size distribution for SSU rRNA gene, GP60 gene and TP14 microsatellite in *Cryptosporidium* spp. isolates from calves and adult cows in Gran Canaria.

Locus and allele size (bp)	Nomenclature	GenBank accession number	Calves		Adults Cows	
			No. of isolates (*n* = 120)	No. of farms (*n* = 15)	No. of isolates (*n* = 120)	No. farms (*n* = 15)
**SSU rRNA gene**						
383	*C. bovis* *C. ryanae*	PP177439 PP177440	7	4	1	1
389	*C. andersoni*	PP177441	0	0	2	2
393–396	*C. parvum*	PP177444	55	14	35	15
**GP60 gene**						
324	*C. parvum*	-	0	0	11	7
337	*C. parvum*	-	1	1	2	2
340	*C. parvum* IIdA16G1	PP333105	9	3	0	0
354	*C. parvum*	-	2	2	4	2
358	*C. parvum* IIdA22G1	PP333106	13	5	1	1
361	*C. parvum* IIdA23G1	PP333107	12	5	3	3
364	*C. parvum* IIdA24G1	PP333108	2	1	0	0
358 + 361	*C. parvum*	-	3	2	0	0
**TP14**						
333 342	*C. parvum* *C. parvum*	PP213048 PP213049	2 31	1 11	0 0	0 0

Three alleles were observed for the SSU rRNA gene marker, with sizes ranging from 383 to 396 bp, depending on the *Cryptosporidium* species. Alleles 393 and 396 were the most common, found in 45.8% (55/120) of calves and 29.2% (35/120) of adults, and were identified in specimens from nearly all farms. Sequence analysis of representative isolates for the SSU rRNA gene confirmed that both alleles corresponded to *C. parvum* and showed 100% identity to multiple *C. parvum* reference sequences deposited in GenBank. The 383 bp allele was identified in a much smaller number of samples from calves (5.8%; 7/120) and in a single sample from adults. Sequencing of representative isolates revealed that this fragment size corresponded to either *C. bovis* or *C. ryanae*, with sequences being identical to isolates from calves previously reported in several countries and deposited in GenBank. Two specimens from different farms showed an allele of 389 bp, which was identified as *C. andersoni* by sequence analysis of the PCR products. This sequence was unique and exhibited two nucleotide polymorphisms (G631A and G634A) compared to the reference sequence JX437080 from calves in the USA.

All PCR-positive GP60 gene products were obtained from isolates with SSU rRNA fragment sizes corresponding to *C. parvum* (393 and 396 bp). Overall, 42 isolates from calves (35%) and 21 isolates from adult cattle (17.5%) tested positive at the GP60 gene locus, which was the most polymorphic marker, exhibiting seven alleles ranging in size from 324 to 364 bp. Three specimens from calves on two different farms displayed CE electropherograms indicative of mixed infections with two alleles (358 and 361 bp). Allele 324 was not detected in specimens from calves, while alleles 340 and 364 were not seen in specimens from adults. Representative isolates for alleles 340, 358, 361, and 364 were successfully amplified, yielding sequences indicative of distinct subtypes within the IId family, which differed by multiples of TCA repeat in the trinucleotide repeat region. Alleles 358 and 361 were the most common among faecal specimens from calves and corresponded to subtypes IIdA22G1 and IIdA23G1, respectively, with each being identified on five different farms. Repeated attempts to sequence the predominant allele in adults (324) and alleles 337 and 354 were unsuccessful. The allele 340 sequence was homologous to reference sequences previously described in humans or animals, while the allele 364 shared only 100% similarity with the EU549714 and OR491781 sequences. In contrast, the sequence for allele 358 exhibited a nucleotide polymorphism (G352A) compared to the reference sequence MH796391 from humans in The Netherlands. The same transition was also observed in the sequence for allele 361.

All PCR-positive products at the TP14 microsatellite identified among specimens from calves (27.5%) had previously been confirmed as *C. parvum* based on PCR results at the SSU rRNA locus. Fragment analysis at TP14 marker revealed only two alleles (333–342 bp), with the former identified in just two isolates from a single farm in the south of the island. Sequences of representative isolates for both alleles matched 100% with GenBank reference sequences JF342562 and JQ954684 from calves and lambs in Spain, respectively.

**Figure 3.**
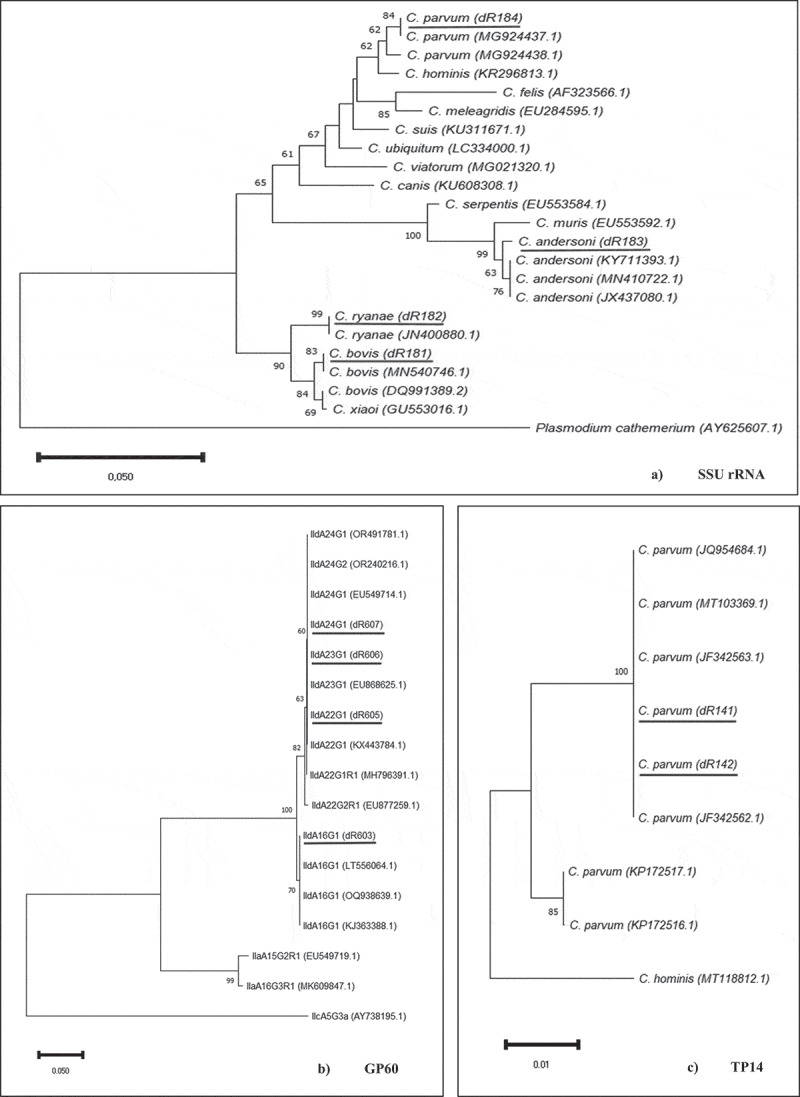
Phylogenetic analysis of the SSU rRNA (a), GP60 (b) and TP14 (c) locus using Neighbour-joining trees based on Kimura two-parameter model. Bootstrap values over 50% from 1000 pseudoreplicates are indicated at the left of the supported node. Scale bar indicates an evolutionary distance of 0.050 (a,b) − 0.01 (c) substitutions per site in the sequence.

Neighbour-joining trees were constructed from aligned SSU rRNA gene, GP60 and TP14 sequences obtained from this study and from those downloaded from the GenBank database (Figure 3a–c). Phylogenetic analysis of sequences from representative isolates at the SSU rRNA gene showed that all species clustered within a well-supported group alongside reference sequences for recognized *Cryptosporidium* spp. Phylogenetic analysis of the GP60 gene demonstrated three well-defined groups based on family: IId, IIa and IIc. As for the phylogenetic analysis of TP14, both sequences from this study clustered together with sequences of *C. parvum* from GenBank.

### 3.3. Questionnaire analysis

#### 3.3.1. Farm characteristics and management data

Six of the 15 farms were considered medium sized (51–200 animals) and the remaining were classified as big farms (>200 animals). All animals sampled were from dairy cattle farms, and 13 of the 15 farms were using a commercial mix to feed the animals, while the remaining two farms also used leftovers of agriculture and banana plantations, and a combination of hay and straw.

All farms sampled had milking parlour and several pens for flock distribution according to production and age, and most of them had an area for artificial lactation, and quarantine facilities. In terms of hygienic care of the facilities, most of the farms removed manure daily or weekly, and disinfection of pavemented areas was generally carried out in a daily basis. In nine farms, no previous parasitological analysis was carried out, while in the other farms, coprologies were performed with intervals of 1 month (1 farm), 6 months (two farms) or once a year (three farms).

Regarding health prevention programmes, most farms vaccinated against three to five diseases, but the use of anticoccidial treatment was not the general trend, with toltrazuril (Baycox®) being the anticoccidial most frequently used. In 67% of the farms no preventive treatment against neonatal diarrhoea was applied, and 12 of the 15 farms reported to have used antibiotics such as marbofloxacin, enrofloxacin and sulfamide/trimethoprim for treating neonatal diarrhoea.

#### 3.3.2. Parasitological knowledge

Only in 4 out of the 15 farms included in the study, the farmers were familiar with *Cryptosporidium*, with two of them having had cryptosporidiosis outbreaks in the past, and another one being owned by a veterinarian. On the other farms sampled, most farmers were unaware of the importance of cryptosporidiosis, linking most of the cases of diarrhoea and neonatal deaths to bacteria such us *Escherichia coli*.

#### 3.3.3. Economic impact

Clinical signs related to *Cryptosporidium* spp. (diarrhoea, dehydration, weight loss, neonatal death, etc.) were pointed out as not frequent by most farmers, and only two farms reported high neonatal mortalities in calves (cryptosporidiosis outbreaks mentioned above). These same two farms were the ones that invested funds in specific treatments for cryptosporidiosis. For other agents of neonatal diarrhoea such as *E. coli, Coronavirus* or *Rotavirus*, the majority of the farms reported spending ≥200 euros per year.

Farmers on all except one farm reported spending more than 30 h per year exclusively on controlling neonatal diarrhoea, while on only six farms the veterinarian noted spending the same time range.

#### 3.3.4. Correlation between questionnaire and parasitological data

Since correlations of surveyed issues with the parasitological parameters had to be done at farm level, and the number of farms was limited, statistical significance was hardly ever obtained. The following are some of the main evidence of tentative correlations recorded.

By analysing the management measures, a relationship between farm size and the number of oocysts excreted was observed in calves, with the highest counts being found in larger farms. In addition, 61.5% of the farms that performed artificial lactation had low-medium mean oocysts counts in calves, while the one that performed natural lactation had high mean counts oocyst. In relation to hygiene-related factors, e.g. manure removing and cleaning of cemented areas, no clear association with the oocyst excretion could be demonstrated.

As for health prevention measures, 49.9% of the farms with a lazaretto for isolation of sick animals had low mean oocyst counts in calves, although farms with lazaretto facilities and high OEC were also detected. By contrast, most farms with lazaretto facilities had no faecal oocysts in adults. On the other side, farms vaccinating against three to five diseases (8) had low mean oocyst counts in calves, while most of the farms vaccinating against less than three diseases had the highest mean oocyst counts. Finally, on farms where neonatal diarrhoea was treated preventively, a mean oocyst count of 0 oocysts was observed in adults, while all farms that were not preventively treated tested positive. However, all farms were positive in calves regardless of whether the animals were treated.

In terms of clinical parameters, only in one farm, in which an outbreak of cryptosporidiosis occurred in the past, the farmer gave a very high importance to the disease, referring a high number of deaths per year and weight losses due to cryptosporidiosis. In this farm, the mean oocyst counts at the time of sampling were low.

Farms spending more funds on specific treatments against neonatal diarrhoea had low or moderate mean oocyst counts in calves, and no oocysts were detected in adults in these farms. Finally, also related with economic issues, there was a negative association between the time invested by farmers and veterinarians to manage neonatal diarrhoea and the amount of oocyst excreted, being the only significant risk factor recorded (*p* < 0.05).

## 4. Discussion

This study determined the occurrence of *Cryptosporidium* spp. in dairy cattle farms in the Spanish island of Gran Canaria, focusing on both suckling calves and adults, while identifying the genetic diversity using a fragment size analysis technique with different markers. The results confirm the high frequency of *Cryptosporidium* infection and the genetic heterogeneity of *C. parvum* subtypes, including zoonotic subtypes. Consistent with previous studies that recommend considering this protozoan in differential diagnosis for neonatal diarrhoea in calves [39,40], the faecal scores of the suckling calves sampled correlated with the intensity of infections. However, as other possible aetiological agents such as bacteria or viruses were not studied, the clinical signs observed cannot be not fully attributed to *Cryptosporidium* spp.

Most studies on ruminant cryptosporidiosis have found higher infection rates in diarrhoeic calves compared to non-diarrhoeic ones, in line with our results [41–44]. In this study, the detection rate by microscopy in calves (45.9%) was higher than in other countries, such as Iran (1.65%), Colombia (26.6%), Egypt (30.2%) and Italy (38.8%), except for one study in Algeria, which reported a frequency of 52.2% [41,45–48]. In Spain, previous studies reported higher detection rates, ranging from 49.2% to 57.8% [10,36,49–51]. Variations in prevalence rates between studies may reflect actual differences in parasite occurrence but can also be attributed to factors such as sample processing, seasonal differences, or the methodologies used for identifying *Cryptosporidium* oocysts. On the other hand, in adult cattle the detection rate by microscopy was very low (4.1%), which aligns with findings by Vergara-Castiblanco et al. [52] using carbol fuchsin staining. In contrast, Lorenzo Lorenzo et al. [53] detected *Cryptosporidium* oocysts in 71.7% of faecal samples from asymptomatic adult cows using Heine’s technique. Weber et al. [54] reported that a 100% detection rate in humans required 500,000 oocysts per gram of faeces, highlighting the low sensitivity of acid-fast stains. Despite the low parasitic load, the large volume of faeces excreted by asymptomatic infected cows may pose a significant risk of environmental contamination for newborn calves, particularly during parturition. Consequently, several authors have recommended using more sensitive and specific techniques for detecting *Cryptosporidium* spp. in ruminants [55–58]. In line with these recommendations, this study found that PCR was significantly more sensitive than Kinyoun staining, particularly in detecting infections in adult animals. These results were in accordance with previously published findings from several studies [48,59–62], contrary to that described by Taha et al. [63], who found a positive sample detection rate of 58.3% and 35.5% by microscopy and PCR analysis, respectively.

The SSU rRNA gene marker identified the presence of four *Cryptosporidium* species previously noted by Xiao [14] as the most prevalent in cattle: *C. parvum*, *C. ryanae*, *C. bovis,* and *C. andersoni*. Among these, *C. parvum* was the most common species in calves, consistent with previous Spanish and European studies [36,45,47,50,57,64]. *C. parvum* was also the predominant species in adults, in contrast to that described in several surveys, showing that *C. andersoni* is commonly the most frequent species of *Cryptosporidium* in adults [65–67]. Therefore, adults would be an important source of infection for newborn calves in this study.

As reported in earlier research, the distribution of *Cryptosporidium* species in cattle followed an age-related infection pattern [1,4,14,68]. *C. andersoni* was only observed in adults, supporting data previously published [69,70]. On another hand, *C. ryanae* and *C. bovis* are mostly found in post-weaned calves [67], although *C. ryanae* has previously been described in pre-weaned calves [57]. Unfortunately, we obtained the same allele size in genotyping for *C. bovis* and *C. ryanae* and did not sequence all samples, preventing differentiation between the two species, just like in Ramo et al. [18]. Interestingly, a positive adult sample corresponding to *C. bovis/C. ryanae* allele size was observed here, suggesting that both species could be found across all age ranges, as reported elsewhere [10].

In the mainland part of Spain, both IIa and IId subtype of *C. parvum* have been described in ruminant hosts, with the subtype family IIa being preferentially reported in calves. The potentially zoonotic subtype IIaA15G2R1 is considered one of the most important due to its widespread dissemination worldwide, while the subtype family IId has been more frequently observed in goat kids and lambs [1,25,36,50]. In this study, GP60 subtyping revealed that most *C. parvum* from cattle farms in Gran Canaria belonged to the subtype family IId, with fragment sizes corresponding to subtypes IIdA22G1 and IIdA23G1 found in approximately half of the fully characterized isolates. Additionally, we reported a high genetic diversity, detecting four different subtypes of the IId family, as well as other fragment sizes that could not be assigned to a specific subtype due to unsuccessful amplification by PCR. This indicates a higher genetic heterogeneity of *C. parvum* isolates in this study compared to those reported in Galicia (north-western Spain), where only two distinct subtypes, IIaA15G2R1 and IIaA13G1R1, were identified in calves [50].

In other European countries such as Portugal, Germany, Hungary, Belgium, Serbia, and Montenegro, the presence of the subtype family IId in calves has been reported, but at a lower prevalence than the subtype family IIa [14]. However, a study in Egypt found that all *C. parvum*-positive samples in dairy calves belonged to subtype family IId, specifically subtype IIdA20G1 [45]. Based on the results of the current study, explaining the high prevalence of subtype family IId in calves is challenging. Discrepancies may arise from geographical variations due to genetic isolation of animals imported in the past with these *C. parvum* subtypes. Additionally, the Canary Islands have historically been characterized by the farming of small ruminants, such as goats, suggesting that *C. parvum* isolates of the IId family may have been introduced in cattle farms. Therefore, further studies involving a larger number of isolates from different geographical regions of the Canarian Archipelago (i.e. other islands), conducted in different seasons and across various management and husbandry systems, are necessary to confirm these observations.

The TP14 microsatellite was the least polymorphic marker, with only two alleles identified in this study, 333 bp (PP213048) and 342 bp (PP213049). The two sequences obtained in this study were found to be identical to those previously published in several Spanish studies [23,25,37], although the frequency of allele distribution was different, with the 333 allele being the predominant allele.

Phylogenetic analyses of the SSU rRNA marker revealed a high degree of similarity between *C. bovis* and *C. ryanae*, as in the studies conducted by Li *et al.* [71] and Zhao *et al.* [72]. Additionally, the close relationship between the isolates examined in this study and some reference isolates previously classified as subtype family IId is demonstrated in the phylogenetic analyses of the *GP60* gene. On the other hand, the phylogenetic analysis of TP14 shows a great genetic similarity between the sequences obtained in this study, and sequences described by Quílez et al. [25,37] in Spain.

The questionnaires completed by farmers during the sampling visits provided valuable information on the management and sanitary measures used on farms throughout the island of Gran Canaria, as well as on the farmers’ knowledge of the diseases responsible for calf scours (including cryptosporidiosis). However, it was difficult to consistently identify risk factors by linking the parasitological results to the questionnaire data for several reasons: (1) the relatively small number of farms included in the study (Gran Canaria has a limited number of cattle farms overall); (2) the wide range of factors that may influence not only the epidemiology but also the endogenous development of the parasite; or (3) the low number of positive samples identified through microscopy, particularly in adults cattle. Nonetheless, despite the lack of statistical significance in most cases, some associations were observed, as discussed below.

The survey data showed that larger farms, and those that used natural lactation, had higher oocyst counts in calves. This suggests that increased contact among animals, particularly between calves and asymptomatic carriers’ dams during lactation, raises the probability of infection, as previously noted by Skerrett and Holland [58]. On the other hand, cleaning frequency and other related hygienic measures did not show a clear negative correlation with oocyst counts. This may be explained by the high resistance of *Cryptosporidium* oocysts to most disinfectants and the low infective dose required for disease development, making it extremely difficult to eliminate the parasite from farms [57,73]. Regarding preventive health measures, the presence of an isolation area (lazaretto) for sick animals was associated with lower infection rates, although this correlation was more pronounced in adult cattle and thus may have limited relevance for cryptosporidiosis in calves. Additionally, farms with a comprehensive vaccination plan tended to have lower oocyst counts, likely because these farms generally had better implementation of sanitary practices.

Surprisingly, most farmers barely noted clinical signs that might be associated with cryptosporidiosis, such as growth delays or diarrhoea, yet some of these farms had high oocyst counts. Conversely, farms where clinical signs of cryptosporidiosis were noted, sometimes accompanied by significant mortality, showed variable oocyst counts. These last farms had experienced severe cryptosporidiosis outbreaks, butdespite the improvement of preventive measures, the results achieved were variable.

Interviews with farmers revealed a general lack of awareness about *Cryptosporidium*, its clinical and pathological impacts, and its economic consequences for livestock production. This knowledge gap likely contributes to insufficient investment in differential diagnostic testing, as many farmers attributed neonatal diarrhoea to colibacillosis. Misdiagnosis cryptosporidiosis could lead to substantial economic losses, not only due to the cost of ineffective treatments but also because specific measures for *Cryptosporidium* prevention and control are often neglected. Furthermore, the unnecessary use of antibiotics can contribute to antibiotic resistance in *E. coli* and other bacterial pathogens [74,75]. On farms that invested in specific treatments for cryptosporidiosis such as halofuginone, lower oocyst counts were detected in both calves and adults. Interestingly, of the four farms that implemented such treatments, two had experienced serious cryptosporidiosis outbreaks, and one was owned by a veterinarian. The fourth farm, despite having a relatively high budget for treating neonatal diarrhoea, had average oocysts counts. This may be due to improper application of prophylactic and therapeutic measures, as there are currently no highly effective treatments for cryptosporidiosis on the market, nor standardized treatment protocols for this disease [1].

In conclusion, this study highlights a high prevalence of *Cryptosporidium* on cattle farms in Gran Canaria, Spain. The most common *Cryptosporidium* species infecting cattle were identified, and molecular detection through PCR proved to be far more sensitive than microscopic observation using Kinyoun staining, particularly in adult samples. The detection of *C. parvum* in a significant number of adult cattle underscores their role as reservoirs for this zoonotic species and as a source of infection for cryptosporidiosis in suckling calves. Fragment typing of the GP60 marker revealed distinct IId subtypes, which is considered an unusual subtype family compared to previous studies on cattle farms in mainland Spain. Further research is needed to determine whether these findings extend to cattle farms in other Canary Islands, and to small ruminant farms. Within the context of zoonotic transmission of *Cryptosporidium*, the involvement of free-ranging animal should be also addressed. Finally, the results of the questionnaires underline the importance of raising awareness among farmers about the importance of bovine cryptosporidiosis, and to further identify risk factors for its control. To the best of the authors’ knowledge, this is the first published description of *Cryptosporidium* species and subtypes in cattle farms on the island of Gran Canaria, Spain.
